# Neuropeptide S Protects Dopaminergic Neurons in a Paraquat-Induced Parkinson’s Model Using SH-SY5Y Cells

**DOI:** 10.1007/s12035-025-05401-7

**Published:** 2026-02-02

**Authors:** Fatma Gonca Koçancı, Mehmet Bülbül, İrem Akçalı, Dijle Kipmen-Korgun, Ebral Çubukçu, Mutay Aydın Aslan, Aleyna Öztüzün, Simla Su Akkan, Tugçe Çeker, Aysel Agar

**Affiliations:** 1 Department of Medical Laboratory Techniques, Vocational High School of Health Services, Alanya Alaaddin Keykubat University, Alanya/Antalya, Türkiye; 2 Department of Physiology, Faculty of Medicine, Akdeniz University, Antalya, Türkiye; 3 Department of Medical Biochemistry, Faculty of Medicine, Akdeniz University, Antalya, Türkiye

**Keywords:** Parkinson’s disease, SH-SY5Y cells, Neuropeptide S, Paraquat, Oxidative stress, Dopaminergic signaling pathways

## Abstract

This study is the first to comprehensively explore both intracellular and computational mechanisms through which Neuropeptide S (NPS) protects against paraquat-induced dopaminergic toxicity in a Parkinson’s disease (PD)-like SH-SY5Y cell model. Paraquat induces oxidative stress, mitochondrial dysfunction, and dopaminergic neuron loss, mimicking key PD features. Bioinformatic analyses, including Reactome pathway mapping and molecular docking, confirmed a high-affinity interaction between NPS and its receptor NPSR1, activating GPCR-associated signaling. NPS treatment restored intracellular dopamine and ATP levels and increased tyrosine hydroxylase (TH) and vesicular monoamine transporter 2 (VMAT) expression. Cell viability was assessed using the MTT assay, while dopamine levels were measured via LC–MS/MS. p-ERK1/2, total ERK1/2, and Nrf2 were quantified by ELISA and western blot. Oxidative stress markers, including TBARS, MAO-A, MAO-B, and COMT, were analyzed by ELISA. Gene expression of Bax, Bcl-2, Caspase-3, Caspase-8, DAT, and VMAT was evaluated by qRT-PCR. TH, c-Fos, and NPSR1 were visualized using immunofluorescence. NPS significantly improved cell viability and restored ATP levels compromised by paraquat exposure. It also reduced TBARS, MAO-B, and COMT levels, reversed paraquat-induced ERK1/2 phosphorylation, and restored Nrf2 and MAO-A expression. Additionally, NPS upregulated the anti-apoptotic marker Bcl-2. Most of these protective effects were abolished in the presence of the NPSR antagonist ML154, indicating a receptor-mediated mechanism of action. In conclusion, NPS was found to attenuate oxidative stress, support mitochondrial and dopaminergic function, and influence apoptosis-related signaling in our cellular model. These findings suggest that targeting the NPS/NPSR1 system may hold therapeutic potential in neurodegenerative diseases such as PD, warranting further in vivo validation.

**Graphical Abstract:**

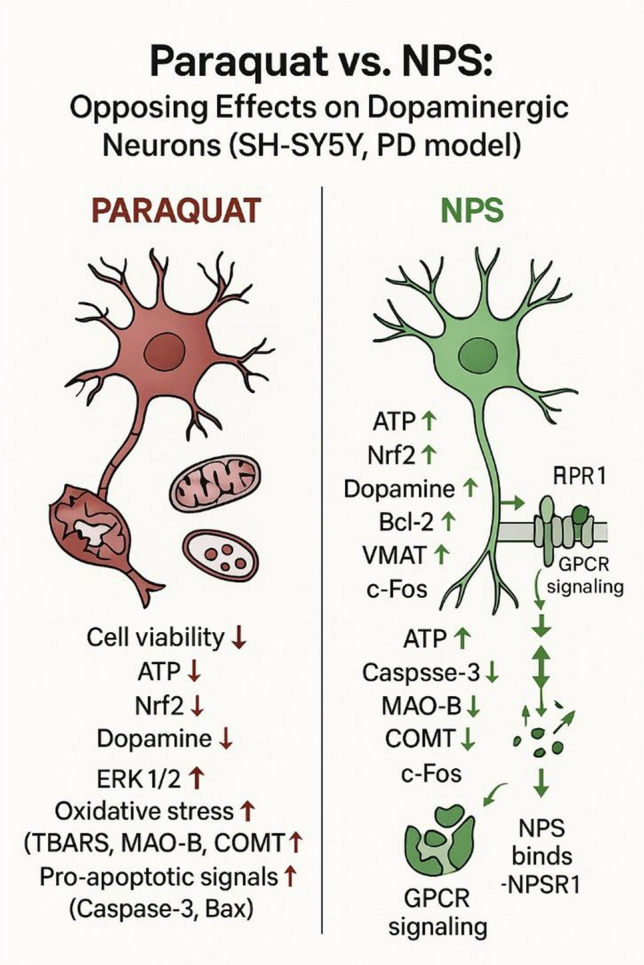

## Introduction

Parkinson’s disease (PD) is one of the most common progressive neurodegenerative disorders of the central nervous system, primarily characterized by the loss of dopamine-producing neurons in the substantia nigra [1]. Clinically, the disease manifests with tremor, rigidity, bradykinesia, and postural instability. Although its pathophysiology has not been fully elucidated, it is widely accepted that oxidative stress and mitochondrial dysfunction play significant roles in the development of PD, which arises from a complex interplay between genetic and environmental factors [2–4].

Current therapeutic strategies mainly provide symptomatic relief but fail to effectively target oxidative damage or halt neurodegenerative progression [5]. Therefore, identifying compounds that can modulate oxidative stress and preserve mitochondrial integrity represents a promising approach for disease modification. In this context, Neuropeptide S (NPS)—a 20-amino acid endogenous peptide identified through reverse pharmacology—has emerged as a promising neuroprotective candidate due to its wide-ranging neuromodulatory and immunoregulatory roles [6, 7]. NPS is primarily expressed in the brainstem, amygdala, and hypothalamus, whereas its receptor, NPSR1, a G protein-coupled receptor, is broadly distributed across the central nervous system. NPSR1 predominantly signals via Gq and Gs pathways, initiating intracellular calcium release, increasing cAMP levels, and activating downstream cascades such as ERK1/2 phosphorylation [8, 9].

In addition to its established functions in regulating arousal, anxiety, and feeding behavior, NPS has been shown to modulate nociceptive processing, influence immune responses through the induction of pro-inflammatory cytokines, and attenuate oxidative stress [6, 10, 11]. NPS also interacts with major neurotransmitter systems—including glutamate, norepinephrine, serotonin, corticotropin-releasing factor (CRF), and GABA—positioning it as a central regulator of synaptic plasticity and neuronal excitability [12]. Furthermore, it enhances dopaminergic transmission by stimulating dopamine release in the ventral tegmental area (VTA) and medial prefrontal cortex, regions associated with reward and executive function [13, 14]. Emerging evidence also implicates NPS in cellular defense mechanisms and neuroinflammation, thereby broadening its functional repertoire beyond neuromodulation [15, 16]. Genetic variants and single nucleotide polymorphisms in the NPSR1 gene have been associated with several pathological conditions, including depression, schizophrenia, asthma, and rheumatoid arthritis, underscoring the pleiotropic nature of this system [6, 17, 18]

Taken together, these findings highlight the multifaceted role of the NPS/NPSR1 system in both central and peripheral mechanisms relevant to neurodegeneration. Its capacity to modulate oxidative stress, immune activation, and dopaminergic signaling renders it a strong therapeutic candidate for neurodegenerative diseases, particularly PD. However, the precise molecular mechanisms through which NPS confers neuroprotection remain to be fully elucidated.

To explore the molecular mechanisms underlying the neuroprotective effects of NPS in PD, we employed the human neuroblastoma SH-SY5Y cell line as an in vitro model. SH-SY5Y cells are widely used in PD-related research due to their capacity to differentiate into dopaminergic neuron-like phenotypes and their expression of critical markers such as tyrosine hydroxylase (TH), dopamine transporter (DAT), and vesicular monoamine transporter 2 (VMAT2). Their documented susceptibility to oxidative damage, especially upon exposure to mitochondrial toxins like paraquat [19], renders them highly suitable for modeling PD-associated dopaminergic degeneration and for evaluating neuroprotective interventions [20, 21].

We assessed whether NPS could mitigate paraquat-induced oxidative injury by evaluating its impact on key molecular targets. These included intracellular ROS levels, lipid peroxidation, and markers of apoptosis such as Bcl-2 (anti-apoptotic), Bax (pro-apoptotic), and caspase-3 (executioner caspase) [22–26]. In addition, we explored the activation of Nrf2, a master regulator of antioxidant response, and the activity of dopamine-metabolizing enzymes monoamine oxidase (MAO) and catechol-O-methyltransferase (COMT), both of which contribute to oxidative stress through dopamine catabolism [27].

To assess mitochondrial function, intracellular ATP levels were measured, providing insight into the bioenergetic status of cells under oxidative challenge and NPS treatment. Restoration of ATP content in paraquat-exposed cells would indicate preservation of mitochondrial function by NPS.

We also measured the expression of dopamine transporters, including dopamine transporter (DAT), which facilitates dopamine reuptake from the synaptic cleft, and vesicular monoamine transporter 2 (VMAT2), which sequesters dopamine into synaptic vesicles to reduce cytosolic ROS generation. Furthermore, to determine whether the observed cellular effects of NPS are mediated specifically through its receptor, we used ML154, a selective antagonist of the neuropeptide S receptor (NPSR1). ML154 effectively inhibits NPS-induced receptor activation. By preventing NPSR1 activation, ML154 served as a valuable pharmacological tool to confirm that the antioxidant and antiapoptotic effects of NPS observed in our study are indeed receptor-dependent [28, 29].

In parallel, we conducted bioinformatic analyses, including Reactome pathway enrichment and molecular docking simulations, to predict the binding affinity of NPS to NPSR1 and to map potential downstream signaling networks. These computational approaches complemented our experimental data and supported a receptor-mediated mechanism involving canonical GPCR pathways.

Through this multifaceted approach combining experimental and in silico methods, we aimed to elucidate the underlying mechanisms by which NPS protects dopaminergic neurons from oxidative damage, providing mechanistic insight into its potential therapeutic relevance in PD.

## Material and Method

### Bioinformatic Validation of NPS–NPSR1 Interaction

To support the experimental findings, a combined approach involving curated pathway mapping and structural docking simulations was employed. The Reactome Pathway Database (https://reactome.org) was queried using the terms “Neuropeptide S” and “NPSR1”. This search identified a curated molecular event titled “Neuropeptide S receptor binds neuropeptide S” (Reactome Stable Identifier: R-HSA-444620), which describes the binding of extracellular NPS (UniProt ID: P0C0P6) to its receptor NPSR1 (UniProt ID: Q6W5P4) at the plasma membrane, triggering downstream intracellular signaling. Furthermore, this interaction is embedded within the broader “GPCR downstream signalling” pathway (Reactome ID: R-HSA-373076), supporting the canonical role of NPSR1 in G protein-coupled signal transduction. To complement this pathway-based validation, we also visualized the three-dimensional structures of NPS, NPSR1, and their docked complex using structural models retrieved from the AlphaFold Protein Structure Database (NPS: AF-P0C0P6-F1-v4; NPSR1: AF-Q6W5P4-F1-v4). The receptor–ligand interface and interaction profiles were analyzed with BINANA 2.2 to provide additional molecular-level insights.

### Cell Cultures and Treatments

The human neuroblastoma SH-SY5Y cell line was obtained from the SAP Institute (Ankara, Turkey). Cells were cultured in Dulbecco’s Modified Eagle Medium-F12 (DMEM-F12; E0500-210, CEGROGEN), supplemented with 10% (v/v) heat-inactivated fetal bovine serum (FBS; BI04-007-1A, BIO.IND.) and 100 U/mL penicillin with 100 µg/mL streptomycin (P0100-790, CEGROGEN), referred to as complete medium. Cultures were maintained at 37 °C in a humidified atmosphere of 95% air and 5% CO_2_, following standard protocols [30]. Upon reaching 80% confluence, cells were harvested, counted, and seeded into collagen-coated 6-, 24-, or 96-well plates (5 × 103 cells/cm2) depending on the experimental requirements [21].

To differentiate SH-SY5Y cells (d-SH-SY5Y), the cells were treated with differentiation medium containing 10 µM retinoic acid (RA) (R2625-50MG, Sigma-Aldrich) prepared in complete medium with 2% FBS. The differentiation process was carried out over 6 days, with medium replacement every 2 days [21, 31].

To determine the optimal concentrations and treatment durations for paraquat (856,177-1G, Sigma-Aldrich), NPS (custom-synthesized by GenScript®, Piscataway, NJ, USA; Catalog No: PE8267), and ML154, cells were exposed to 250–2000 μM paraquat, 0.05–2 μM NPS, or 0.1–100 μM ML154 for 24, 48, and 72 h. For the experiments investigating the protective effects of NPS against paraquat-induced toxicity, treatments were applied simultaneously: cells were co-treated with paraquat and NPS (with or without ML154) for the indicated durations. Experimental groups were organized as follows:

1. Untreated control group.
2. NPS-treated group.
3. Paraquat-treated group.
4. Paraquat + NPS-treated group.
5. Paraquat + NPS + 0.1 µM ML154-treated group.
6. Paraquat + NPS + 1 µM ML154-treated group.

### Cell Viability Assay

Cell viability was assessed using the MTT assay (3-[4,5-dimethylthiazol-2-yl]−2,5-diphenyltetrazolium bromide; SE2039502, Serva) as described previously [32]. SH-SY5Y cells were seeded into collagen-coated 96-well plates at a density of 5 × 10^3^ cells/cm^2^ and allowed to adhere for 24 h before treatment. After completing differentiation and treatment procedures [21], 10 µL of 5 mg/mL MTT solution was added to each well, followed by incubation for 4 h at 37 °C and 5% CO_2_. Supernatants were then removed, and the resulting formazan crystals were dissolved in 200 µL of DMSO (SE3975702, Serva) per well. Absorbance was measured at 570 nm and 690 nm using a microplate reader (Bio-Tek Instruments, Inc.), and cell viability was expressed as a percentage relative to untreated controls, which were set at 100%.

### Validation of the In Vitro Parkinson Model

To validate the cellular PD model, immunocytochemical staining was performed using antibodies against tyrosine hydroxylase (TH) and ubiquitin C-terminal hydrolase-L1 (PGP9.5), markers of dopaminergic/noradrenergic neurons and general neuronal structure, respectively. SH-SY5Y cells were seeded at a density of 5 × 10^3^ cells/cm^2^ on collagen-coated six-well plates and divided into three groups: untreated control, RA-differentiated for 6 days, and RA-differentiated followed by 1000 μM paraquat exposure for 24 h. Cells were fixed with 4% paraformaldehyde for 15 min at room temperature, permeabilized with 0.1% Triton X-100 for 15 min, and blocked with 3% BSA in 0.5% Tween-20/PBS for 30 min.

A primary antibody cocktail containing anti-TH (ab112, Abcam) and anti-PGP9.5 (ab8189, Abcam) was diluted 1:1000 in PBS and incubated overnight at 4 °C. After washing, cells were incubated with goat anti-rabbit IgG (A11034, Invitrogen) and goat anti-mouse IgG (A11003, Invitrogen) secondary antibodies (1:200 in PBS) for 1 h at room temperature. Nuclei were counterstained with DAPI. Fluorescence was visualized using filters for DAPI (I3), FITC (N2.1), and Texas Red at 40 × magnification.

Neurite length analysis was performed using ImageJ software (NIH, USA) on at least 50 cells per group, selected from multiple non-overlapping microscopic fields to ensure unbiased sampling. Neurite length was measured from the cell body to the tip of the longest neurite; shared neurites were assigned to a single cell. Data were expressed as mean ± standard deviation.

### Assessment of Mitochondrial Function: Intracellular ATP Content

Mitochondrial function was evaluated by measuring intracellular ATP levels in all experimental groups. d-SH-SY5Y cells were cultured in 60-mm dishes and formed into experimental groups. For ATP quantification, cells were rinsed with cold PBS and lysed using the lysis buffer provided in a commercial ATP determination kit (ab113849, Abcam Cambridge, UK). The lysates were centrifuged at 10,000 × g for 10 min at 4 °C, and the supernatants were transferred to a white 96-well plate. Luciferin-luciferase reagent was added to initiate the bioluminescent reaction, and luminescence was recorded using a microplate reader. ATP concentrations were calculated from a standard curve and normalized to total protein levels, which were determined by the Bradford assay.

### ERK and Nrf2 Signaling Assay

ERK1/2 phosphorylation levels and Nrf2 signaling activity were assessed using the InstantOne ELISA Total/Phospho ERK1/2 kit (Invitrogen, Thermo Fisher Scientific; Catalog No. 85–86013-11) and the Human NRF2 ELISA kit (Invitrogen, Thermo Fisher Scientific; Catalog No. 17184643), respectively, according to the manufacturers’ instructions.

### Western Blot Analysis

SH-SY5Y cells were seeded into collagen-coated six-well plates at a density of 5 × 103 cells/cm2. Following differentiation and treatments, cells were washed with ice-cold PBS and lysed on ice using radioimmunoprecipitation assay (RIPA) buffer (39,244, Serva) supplemented with protease inhibitors (P2714-1BTL, Sigma) and phosphatase inhibitors (PhosSTOP, 04906845001, Roche). Lysates were collected by scraping and centrifuged at 14,000 × g for 15 min at 4 °C to remove cellular debris. Protein concentrations were determined using the Pierce BCA Protein Assay Kit (44,132, Expedeon). Equal amounts of protein (30 μg per lane) were separated by 10% SDS-PAGE and transferred onto nitrocellulose membranes. Membranes were blocked with 5% non-fat dry milk in Tris-buffered saline containing 0.1% Tween-20 (TBST) for 1 h at room temperature. Subsequently, membranes were incubated overnight at 4 °C with primary antibodies against p-ERK1/2 (1:1000, AF1015, Affinity), ERK1/2 (1:1000, AF0155, Affinity), and Nrf2 (1:500, NB100-91897, Novus Biologicals). After washing, membranes were incubated with horseradish peroxidase (HRP)-conjugated secondary antibodies (1:10,000, BA1054, Boster) for 1 h at room temperature. Protein bands were visualized using an enhanced chemiluminescence (ECL) substrate and detected with a Fluorchem Imaging System (G:BOX Chemi XRQ). β-actin (1:3000, AF7018, Affinity) was used as a loading control. Densitometric analysis of band intensities was performed using GeneSys image acquisition software.

### Measurement of MAO-A, MAO-B, COMT, and TBARS Levels in SH-SY5Y Cells

The concentrations of MAO-A, MAO-B, COMT, and Thiobarbituric acid reactive substances (TBARS) in human neuroblastoma SH-SY5Y cells were determined using commercially available enzyme-linked immunosorbent assay (ELISA) kits (MAO-A: E1984Hu, MAO-B: E3822Hu, COMT: E3731Hu, TBARS: E3642Hu; Bioassay Technology Laboratory, Jiaxing, Zhejiang, China) according to the manufacturer’s instructions.

Briefly, cell pellets obtained from the experimental groups were resuspended in 1000 µL of PBS and subjected to sonication to ensure complete cell lysis. The lysates were subsequently centrifuged at 3000 rpm for 20 min at 4 °C to collect the supernatant, which was used for subsequent measurements.

For each analyte, appropriate volumes of the supernatants were transferred into microtiter wells in duplicate, followed by incubation steps as described in the kit protocols. Optical densities were measured at 450 nm using a microplate spectrophotometer. Concentrations of MAO-A, MAO-B, and COMT were calculated from their respective standard curves and expressed as pg/mg or ng/mg protein, while TBARS levels were expressed as nmol/mg protein.

### Quantitative Measurement of Dopamine in SH-SY5Y Cells by LC–MS/MS

The dopamine content in SH-SY5Y cell lysates was quantified using liquid chromatography coupled with tandem mass spectrometry (LC–MS/MS) based on a previously described method [33] with slight modifications.

Cell lysates were prepared by adding 250 µL of formic acid buffer to the cell pellets, followed by 30 s of sonication to homogenize the samples. The homogenates were centrifuged at 18,000 × g for 20 min at 4 °C. The resulting supernatants were transferred into LC–MS/MS vials for analysis.

Dopamine standard solutions were prepared by weighing 0.01 g of dopamine (Sigma-Aldrich, St. Louis, MO, USA) into a 10-mL glass tube, dissolving it in 1 mL of 98–100% formic acid, and diluting to 10 mL with LC–MS-grade water.

Quantification was carried out using an ultra-fast liquid chromatography system (LC-20AD UFLC XR, Shimadzu Corporation, Japan) coupled to a triple quadrupole mass spectrometer (LCMS-8040, Shimadzu Corporation, Japan). Chromatographic separation was achieved on an Inertsil ODS-4 column (3 × 100 mm, 2 µm particle size; GL Sciences Inc., Tokyo, Japan) maintained at 25 °C.

The mobile phase consisted of Solvent A (0.1% formic acid and 1% acetonitrile in water) and Solvent B (0.1% formic acid in acetonitrile). A gradient elution program was applied as follows: 5% B to 50% B (0–1 min), 50% B to 95% B (1–2 min), held at 95% B (2–3 min), and returned to 5% B (3–4 min). The flow rate was set at 0.4 mL/min, with an injection volume of 5 µL per sample.

Dopamine detection was optimized under positive electrospray ionization (ESI) mode using multiple reaction monitoring (MRM). The precursor-to-product ion transition for dopamine was set at m/z 155 → 136.9. The retention time for dopamine was approximately 2.51 min. The method exhibited a linear response over a concentration range of 50–1000 ng/mL with a total analysis time of 5 min per sample.

### Quantitative Real-Time Polymerase Chain Reaction (qRT-PCR)

Total RNA was extracted from SH-SY5Y cells using Trizol reagent (15596026, Invitrogen, Thermo Fisher Scientific) according to the manufacturer’s instructions. Following the addition of 1 mL Trizol, cells were scraped, and RNA isolation was completed. The RNA concentration was determined spectrophotometrically at 260 and 280 nm, and RNA samples were stored at − 80 °C.

One microgram of RNA was treated with 1 U of DNase I (Invitrogen, Thermo Fisher Scientific) to remove genomic DNA contamination.

Complementary DNA (cDNA) synthesis was performed using the SuperScript™ III First-Strand Synthesis System Kit (12574026, Invitrogen, Thermo Fisher Scientific). A reaction mixture containing 10 × RT buffer, 25 mM MgCl₂, 10 mM dNTP mix, 0.1 M DTT, 50 ng/μL random hexamers, RNase OUT, and SuperScript III Reverse Transcriptase (200 U/µL) was incubated with 1 µg of DNase-treated RNA. Incubation conditions were 25 °C for 10 min, 50 °C for 50 min, and 85 °C for 5 min.

Quantitative PCR was conducted using specific primers for human **Bax**, **Bcl-2**, **Caspase-3**, **Caspase-8**, **VMAT**, and **DAT**, and the reference gene **GAPDH** (**Bax** F: 5′-CCTTTTGCTTCAGGGTTTCATCC-3′, R: 5′- CAGCTTCTTGGTGGACGCAT-3′; **Bcl-**2 F: 5′-GATTGATGGGATCGTTGCCT-3′, R: 5′-GTCTACTTCCTCTGTGATGTTGTAT-3′, **Caspase-3** F: 5′-CTGCCGTGGTACAGAACTGG-3′, R: 5′-GTCGGCCTCCACTGGTATTT-3′, **Caspase-8** F: 5′-AGGAAAGGGTGGAGCGGATTA-3′, R: 5′-GAACTTGAGGGAGGCCAGAT-3′, **VMAT** F: 5′-GTCCCCATCATCCCAAGTTATC-3′, R: 5′-TAGGAGAAGATGCTCTGGAAGC-3′, **DAT** F: 5′-TACAAAAATGGTGGCGGTGC-3′, R: 5′-GATCTTCCAGACACCAGCGG-3′, **GAPDH** F: 5′-AGGTCGGAGTCAACGGATTT-3′, R: 5′-GATGGCAACAATATCCACTTTACCA-3′). Reactions were performed with Power SYBR® Green Master Mix (4309155, Applied Biosystems) on a QuantStudio 3 Real-Time PCR System (Applied Biosystems). The relative expression of target genes was calculated using the 2^ − ΔΔCT method, with GAPDH as the normalization control.

### Immunofluorescence Staining for Receptor Localization and Neuronal Activation

To evaluate receptor distribution and activity-dependent responses, two immunolabeling strategies were applied.

(i) NPSR1 and c-Fos staining: differentiated SH-SY5Y cells were immunolabeled with rabbit anti-NPSR1 (1:200; Merck, ABN12) and mouse anti-c-Fos (1:500; Genetex, GTX60996) **on** separate coverslips across experimental groups. For positive control of neuronal activation, cells were depolarized with 100 mM KCl prior to fixation to induce c-Fos expression.

(ii) TH and c-Fos double-labeling: to determine activity-dependent responses specifically within dopaminergic-like neurons, coverslips were co-stained with rabbit anti-TH (1:1000; Abcam, ab113) and mouse anti-c-Fos (1:500; Genetex, GTX60996) following paraquat and/or NPS treatment.

Cells were fixed with 4% paraformaldehyde for 15 min at room temperature, washed with PBS, and blocked with UV block (Thermo Scientific) for 1 h. Primary antibodies were incubated overnight at 4 °C, followed by species-specific, highly cross-adsorbed secondary antibodies: donkey anti-rabbit CF555 (1:500; Biotium, 20038–1) and donkey anti-mouse Alexa Fluor 488 (1:500; Invitrogen, A21202)**.** Nuclei were counterstained with DAPI and mounted in antifade medium. Negative controls (omission of primary antibodies) and single-label controls were performed in parallel to validate specificity and exclude spectral bleed-through. Images were acquired with an Olympus BX43 fluorescence microscope under identical magnification and exposure settings, and scale bars (20–50 µm) were included on all images.

#### Statistics

Data were analyzed using GraphPad Prism 7 software (GraphPad Software, USA). Values are presented as mean ± SEM. For normally distributed variables, one-way analysis of variance (ANOVA) followed by Tukey’s post hoc test was applied. Non-normally distributed data were analyzed using the Kruskal–Wallis test followed by the Mann–Whitney U test. Immunofluorescence results were assessed using the Kruskal–Wallis test with Dunn’s post hoc test. Differences were considered statistically significant at *p* < 0.05.

## Results

### In Silico Evidence for NPS–NPSR1 Binding and Signaling

**Fig. 1:**
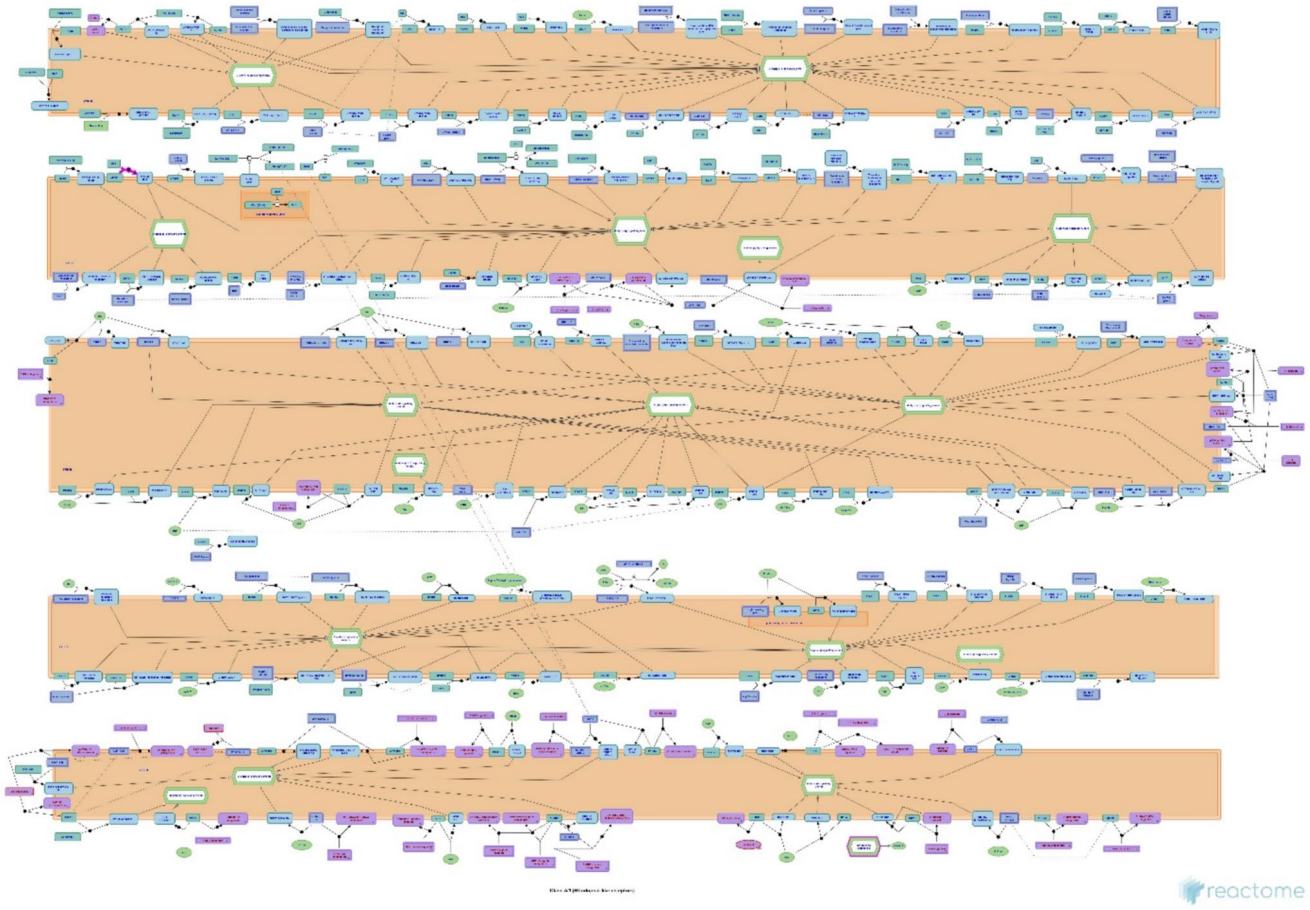
Bioinformatic schematic of NPSR1-mediated GPCR signaling cascade. The figure illustrates the intracellular pathways activated upon the binding of Neuropeptide S to its receptor (NPSR1), as curated in the Reactome pathway R-HSA-373076. Key components include G protein-coupled activation, calcium mobilization, and ERK phosphorylation, which collectively contribute to neuroprotective responses

**Fig. 2:**
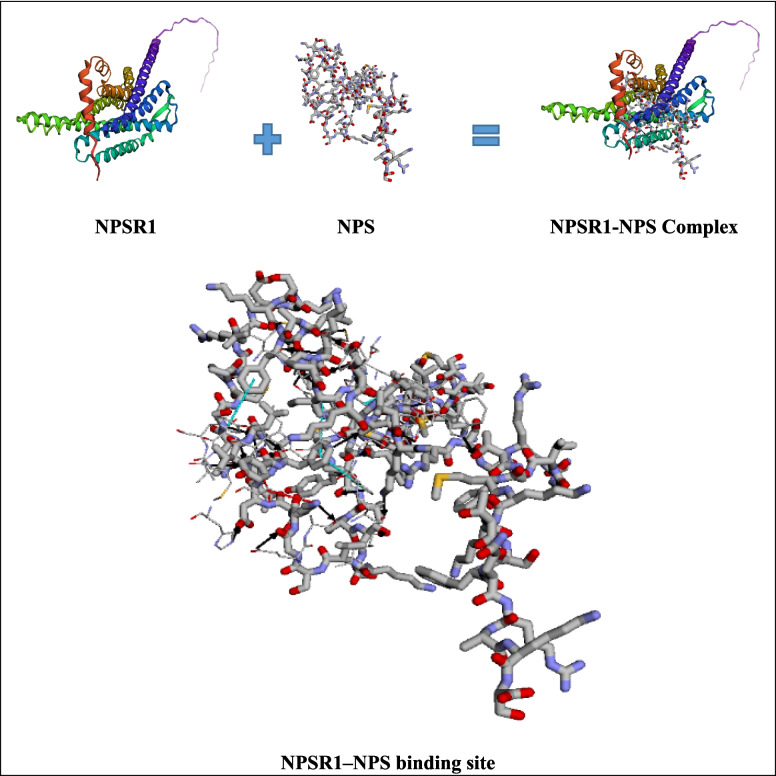
Structural representation of NPSR1, NPS, NPSR1–NPS complex, and a magnified view of the binding site within the complex. Hydrogen bonds are indicated by solid black arrows (donor to acceptor), salt bridges are shown as red dashed lines, and π–π stacking interactions are represented by turquoise dashed lines. Because not all interactions are visible in a single orientation, panel highlights representative binding interactions at higher magnification

The reactome analysis confirmed that NPS specifically binds to its cognate receptor NPSR1 at the plasma membrane, as documented under the molecular event R-HSA-444620. This interaction triggers GPCR-mediated intracellular signaling mechanisms, including Gαq-mediated activation of phospholipase C, mobilization of intracellular calcium stores, and ERK1/2 phosphorylation. These processes are detailed in the “GPCR downstream signalling” pathway map (R-HSA-373076), and provide a mechanistic framework supporting the role of NPS in neuronal modulation and potential neuroprotective functions. A schematic representation of this signaling cascade is shown in Fig. 1. Also, three-dimensional models of the NPSR1, NPS, and the NPSR1–NPS complex are illustrated to demonstrate the receptor–ligand interaction in Fig. 2. The resulting NPSR1–NPS complex revealed a network of non-covalent interactions that likely stabilize ligand binding. Hydrogen bonds, represented by solid black arrows from donor to acceptor, were prominently observed at key contact sites within the binding pocket. Additionally, red dashed lines indicate the formation of salt bridges, contributing to the electrostatic stabilization of the complex. T-stacking interactions, depicted by turquoise dashed lines, were also identified, suggesting π-π interactions between aromatic residues and the ligand. These structural features collectively support the specificity and stability of the NPS–NPSR1 interaction, providing insight into the molecular basis of receptor activation.

### Cell Viability Assay

The dose- and time-dependent cytotoxic effect of paraquat (50–2000 μM) on d-SH-SY5Y cells was evaluated using the MTT assay. The results revealed that 24-h application of paraquat treatment significantly reduced cell viability at 1000 μM and 2000 μM concentrations compared to the control group (65 ± 3%, 48 ± 4% cell viability, respectively) (*p* < 0.05), while lower concentrations did not cause any significant effect. A high degree of cytotoxicity (≥ 50% cell death) was observed at ≥ 1000 μM paraquat after 48 and 72 h of exposure, while lower concentrations did not cause significant cytotoxicity. For subsequent mechanistic experiments, 1000 μM paraquat applied for 24 h was chosen as the working concentration, since it consistently induced robust but sub-lethal toxicity (cell viability > 50%). This dose provided an optimal balance between producing Parkinson’s-like cellular stress and preserving sufficient viable cells for reliable downstream biochemical and immunocytochemical analyses.

The cytoprotective effect of NPS against paraquat-induced cytotoxicity in d-SH-SY5Y cells was also evaluated. The effects of 24 h of application of 0.1–2 μM NPS with 1000 μM paraquat were investigated on d-SH-SY5Y cells. NPS concentrations ranging from 0.1 to 0.5 μM partially restored cell viability (72 ± 4% to 82 ± 5% viability, respectively) compared to the paraquat-only group (67 ± 5% viability). However, concentrations ≥ 1 μM were found to be highly toxic to the cells (Data not shown). The subsequent experiments were carried out using a 24-h treatment with 0.5 mM NPS, which effectively reversed the cytotoxic effects of paraquat.

**Fig. 3:**
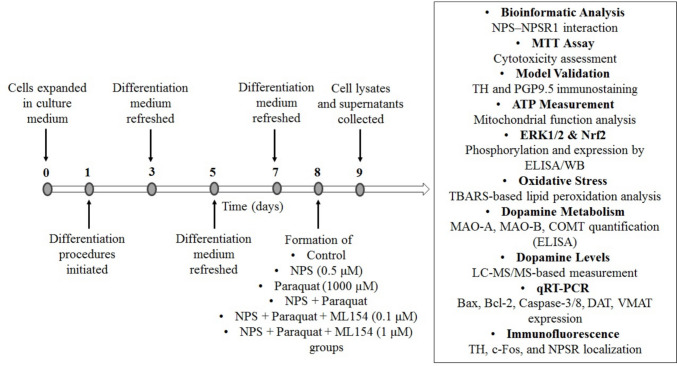
Schematic representation of the experimental timeline based on MTT assay results, illustrating the schedule of differentiation, treatment applications, and sample collection in SH-SY5Y cells

The effect of ML154, an NPS receptor antagonist, on cell viability was determined across a concentration range of 0.1–100 μM for 24 h. ML154 at concentrations ≥ 5 μM caused a statistically significant cell viability decrease (79 ± 4% to 5 ± 2% cell viability) (*p* < 0.05), while ML154 at 0.1–1 μM (99 ± 3 to 92 ± 4% cell viability) did not cause a significant change in cell viability compared to the control group (*p* > 0.05). When SH-SY5Y cells were co-treated with paraquat, NPS, and ML154, a significant reduction in cell viability was observed for ML154 concentrations ≥ 5 μM compared to the NPS (0.5 μM) + paraquat (1000 μM) group (*p* < 0.05). ML154 at 1 μM did not significantly alter cell viability compared to the NPS (0.5 μM) + paraquat (1000 μM) group (*p* > 0.05) (Data not shown). These findings suggest that ML154 at higher concentrations can counteract the protective effects of NPS. Subsequent experiments were conducted with 0.1 and 1 mM ML154 treatments. Based on the results obtained from the MTT analyses, the timeline of subsequent experimental procedures is illustrated in Fig. 3.

### Validation of the In Vitro Parkinson Model

To validate the establishment of an in vitro PD model, SH-SY5Y cells were first differentiated using RA, followed by paraquat-induced degeneration. Dopaminergic neuronal identity was assessed via immunohistochemical detection of TH and PGP9.5.

**Fig. 4:**
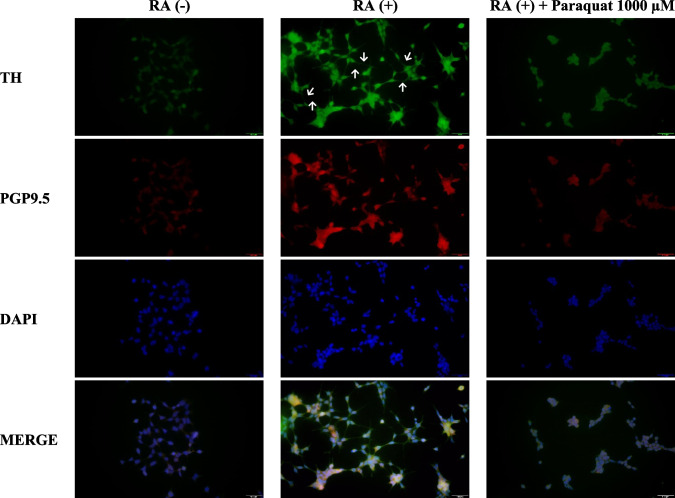
Immunohistochemical detection of TH and PGP9.5 in SH-SY5Y cells cultured under three conditions: undifferentiated control (RA −), differentiated with retinoic acid for 6 days (RA +), and differentiated followed by paraquat exposure (RA + + Paraquat). White arrows indicate neurite outgrowth. Scale bar: 100 μm; magnification: 20 ×

As shown in Fig. 4, RA treatment for 6 days significantly increased TH and PGP9.5 expression in SH-SY5Y cells compared to the untreated control group, confirming successful neuronal differentiation. Additionally, quantitative neurite outgrowth analysis demonstrated a significant increase in the average neurite length in RA-treated cells versus control cells (*p* < 0.05), further supporting the differentiation outcome.

**Fig. 5:**
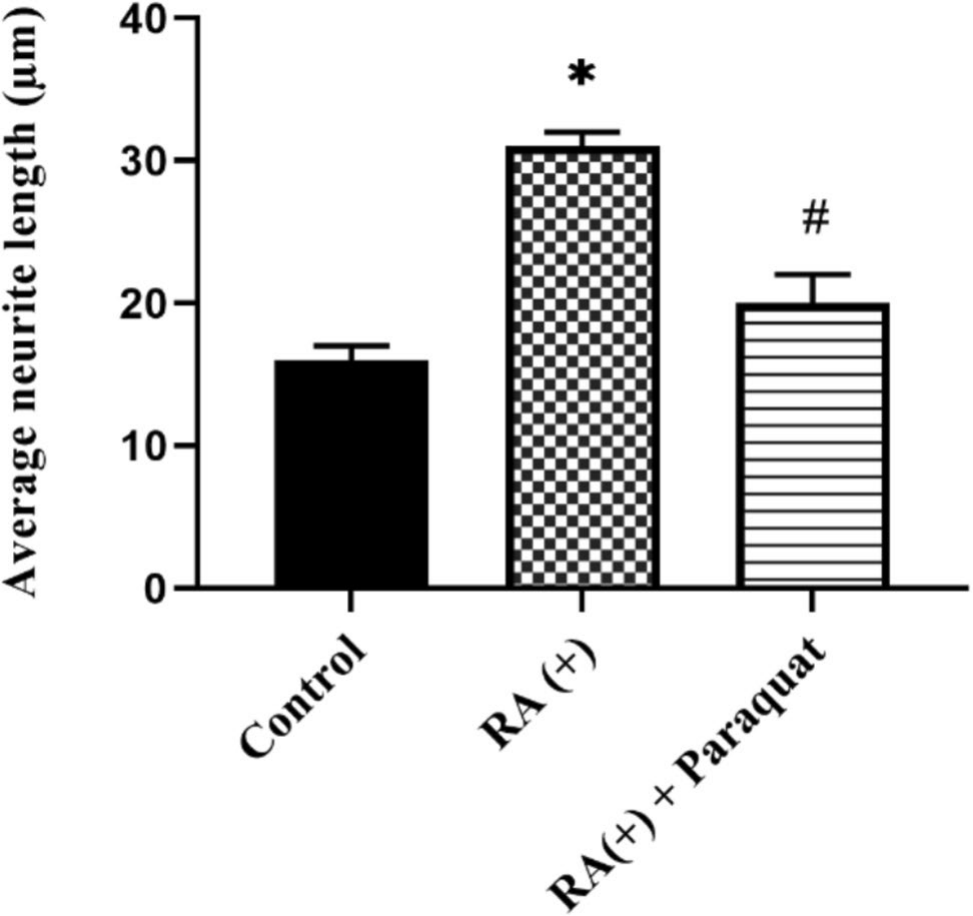
Quantification of average neurite length in SH-SY5Y cells across three experimental conditions: control (RA −), differentiated with retinoic acid for 6 days (RA +), and differentiated followed by paraquat exposure (RA + + Paraquat). (*p < 0.05, compared to the control group without RA, ^#^*p* < 0.05, compared to the group that received only RA). Data are expressed as mean ± SD

Following paraquat exposure (1000 μM for 24 h), a marked reduction in both neuronal marker expression and neurite length was observed, indicating neurodegeneration in the differentiated cells. This effect is quantitatively represented in Fig. 5, where the average neurite length significantly decreased in the paraquat-treated group compared to the RA-only group (*p* < 0.05). Collectively, these findings confirm the successful establishment of an in vitro Parkinson-like neurodegeneration model.

### ATP Levels in d-SH-SY5Y Cells

As shown in Table 1, paraquat exposure led to a significant reduction in ATP levels (53 ± 5% of control) in d-SH-SY5Y cells (*p* < 0.001). Treatment with NPS alone did not alter ATP levels (104 ± 5%) compared to the control group, indicating no mitochondrial toxicity. Co-treatment with NPS and paraquat significantly restored ATP levels (68 ± 3%) relative to the paraquat group (*p* < 0.01), suggesting a protective role of NPS against paraquat-induced mitochondrial dysfunction. However, the co-administration of the NPS receptor antagonist ML154 dose-dependently attenuated these protective effects. At 1 μM ML154, ATP levels (54 ± 4%) declined to values comparable with those observed in the paraquat-only group (*p* < 0.05). These findings indicate that the protective effects of NPS on mitochondrial energy metabolism are likely mediated through NPSR activation and can be reversed by receptor antagonism.

**Table 1:** Quantitative analysis of ATP levels following treatment with paraquat, NPS and ML154 in d-SH-SY5Y cells

	ATP level (% of control)
Control	100 ± 4
NPS	104 ± 5
Paraquat	53 ± 5^#^
Paraquat + NPS	68 ± 3^$^
Paraquat + NPS + 0.1 μM ML154	62 ± 5
Paraquat + NPS + 1 μM ML154	54 ± 4^ϕ^

^#^*p* < 0.05 compared to NPS

^$^*p* < 0.05 compared to Paraquat

^ϕ^*p* < 0.05 compared to NPS + Paraquat-treated group) (*n* = 3)

### ERK1/2 and Nrf2 Levels in d-SH-SY5Y Cells

**Fig. 6:**
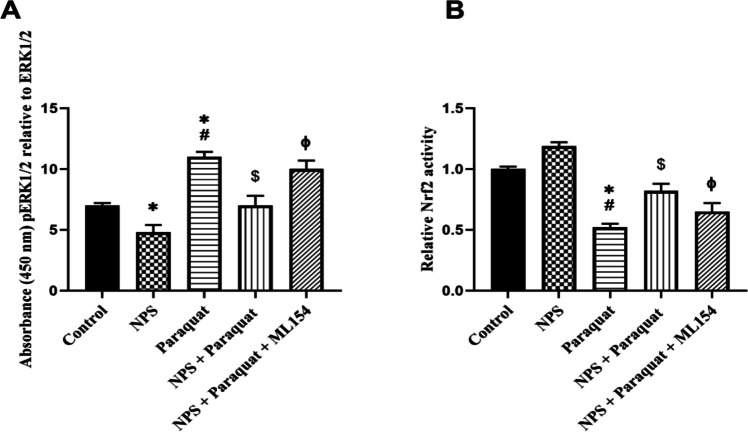
ERK1/2 (**A**) and Nrf2 (**B**) signaling pathways were evaluated in d-SH-SY5Y cells using ELISA assays (*p < 0.05 compared to control, ^#^*p* < 0.05 compared to NPS, ^$^*p* < 0.05 compared to Paraquat, ^ϕ^*p* < 0.05 compared to NPS + Paraquat-treated group) (*n* = 6)

As shown in Fig. 6A, paraquat treatment significantly increased ERK1/2 phosphorylation compared to the control group (*p* < 0.05), indicating activation of this stress-related signaling pathway. Interestingly, while NPS alone reduced pERK1/2 levels below control values (*p* < 0.05), co-treatment with NPS and paraquat significantly attenuated the paraquat-induced increase in ERK1/2 phosphorylation (*p* < 0.05). However, the addition of ML154, a known antagonist, partially reversed this attenuation, leading to a significant re-elevation of pERK1/2 levels (*p* < 0.005 vs. NPS + Paraquat).

In parallel, Nrf2 activity exhibited an inverse pattern (Fig. 6B). Paraquat alone significantly suppressed Nrf2 activity compared to the control (*p* < 0.05), whereas NPS treatment alone slightly enhanced it. Co-treatment with NPS and paraquat partially restored Nrf2 activity (*p* < 0.05 vs. paraquat alone), suggesting a protective effect. However, the inclusion of ML154 in the NPS + paraquat group abolished this restoration (*p* < 0.001), further supporting the regulatory role of NPS on Nrf2 signaling. Results of western blot analysis also support these results.

**Fig. 7:**
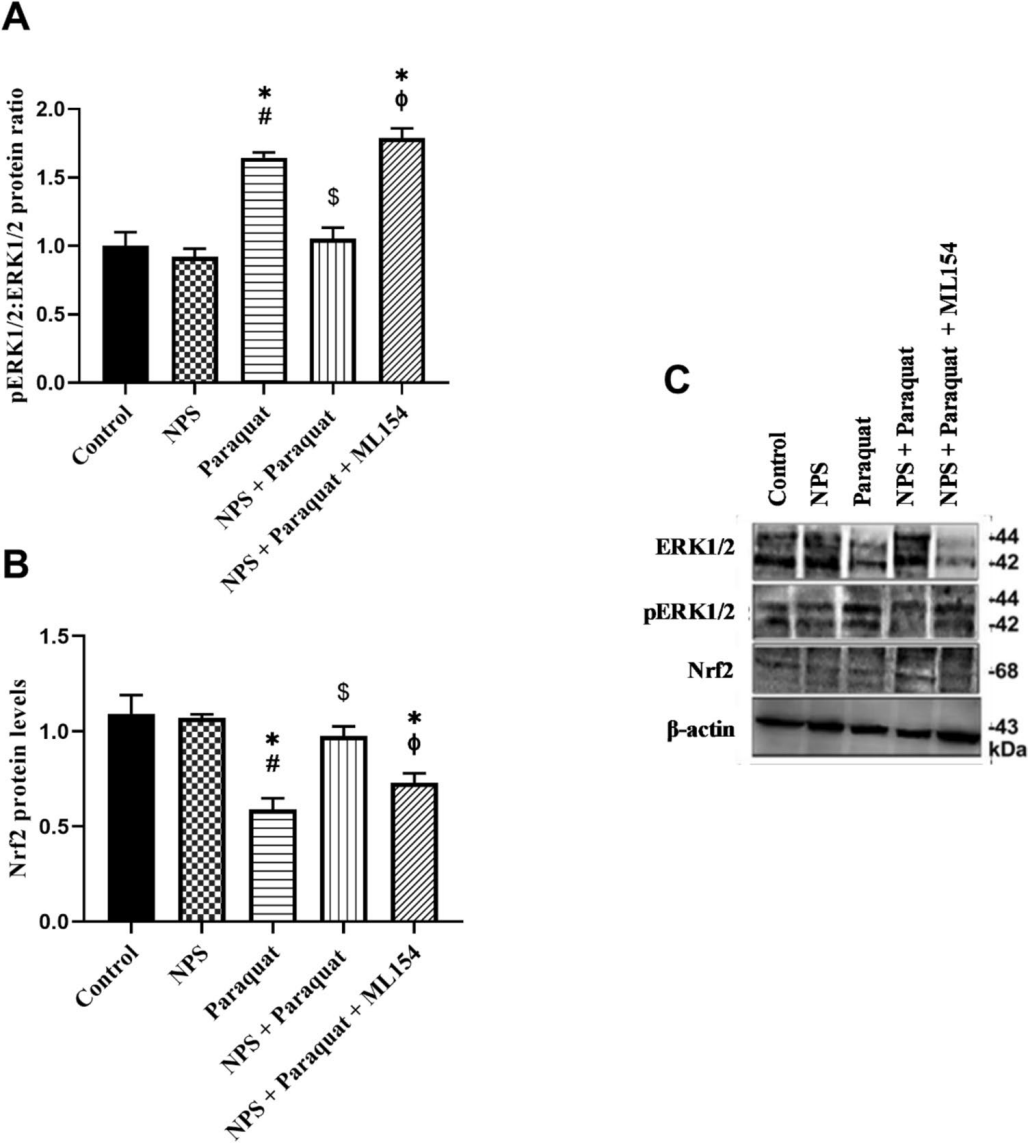
**A**. pERK1/2:ERK1/2 protein ratios, **B**. Nrf2 protein levels, **C**. Blotting image of ERK1/2, p-ERK1/2, Nrf2, and β-actin in d-SH-SY5Y cells. (*p < 0.05, compared to control, ^#^*p* < 0.05 compared to NPS, ^$^*p* < 0.05 compared to Paraquat, ^ϕ^*p* < 0.05 compared to NPS + Paraquat-treated group) (*n* = 3)

As shown in Fig. 7A, C, paraquat treatment significantly increased the p-ERK1/2:ERK1/2 ratio by approximately 1.6-fold compared to the untreated control group (*p* < 0.05). Treatment with NPS alone did not significantly alter this ratio compared to control cells (*p* > 0.05). Notably, co-treatment with NPS and paraquat resulted in a marked reduction (~ 34%) in the paraquat-induced p-ERK1/2:ERK1/2 ratio (*p* < 0.05), suggesting a modulatory effect of NPS on ERK1/2 activation. However, the addition of ML154 (1 μM) to the paraquat + NPS regimen significantly reversed this reduction, restoring the p-ERK1/2:ERK1/2 ratio to levels comparable with those observed in the paraquat-only group (*p* < 0.05).

Regarding Nrf2, paraquat exposure led to a significant decrease (~ 46%) in protein levels relative to the control group (*p* < 0.05; Fig. 7B, C). Co-treatment with NPS significantly restored Nrf2 levels compared to paraquat alone (*p* < 0.05), indicating a potential protective role. Nevertheless, the addition of ML154 to the NPS + paraquat treatment significantly diminished Nrf2 expression (*p* < 0.05), supporting the involvement of the targeted pathway in NPS-mediated effects.

### MAO-A, MAO-B, COMT and TBARS Levels in d-SH-SY5Y Cells

**Fig. 8:**
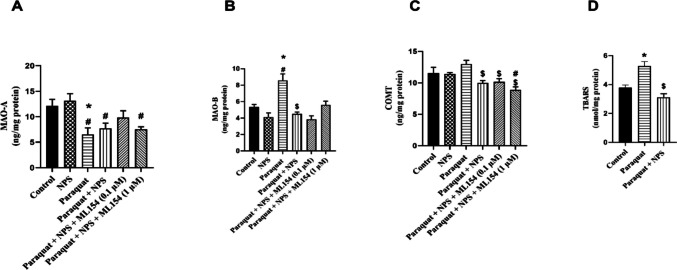
The levels of MAO-A (**A**), MAO-B (**B**), COMT (**C**), TBARS (**D**) were measured in d-SH-SY5Y cell homogenates. (**A** and **B**) *p < 0.05, compared with control, ^#^*p* < 0.05, compared with NPS, ^$^*p* < 0.05 compared with Paraquat-treated group (*n* = 6)

MAO-A levels in the control group were measured as 12.15 ± 1.26 ng/mg protein, while the NPS-treated cells showed comparable levels (13.11 ± 1.42 ng/mg protein), indicating no significant change (*p* > 0.05). Paraquat treatment significantly reduced MAO-A levels to 6.54 ± 1.26 ng/mg protein, consistent with a loss in cellular integrity (*p* < 0.05). Co-treatment with Paraquat + NPS increased MAO-A levels to 7.74 ± 1.02 ng/mg protein. Addition of ML154 at 0.1 µM further elevated levels to 9.85 ± 1.32 ng/mg protein, whereas ML154 at 1 µM slightly reduced them to 7.53 ± 0.54 ng/mg protein (*p* < 0.05). These findings suggest that NPS partially mitigates paraquat-induced reductions in MAO-A levels, likely due to its cytoprotective effects (Fig. 8A).

MAO-B levels in the control group were 5.34 ± 0.52 ng/mg protein, with a reduction observed in NPS-treated cells (4.12 ± 0.49 ng/mg protein). In contrast, paraquat exposure significantly elevated MAO-B levels to 8.59 ± 0.79 ng/mg protein, correlating with increased dopamine degradation (*p* < 0.05). Co-treatment with Paraquat + NPS decreased MAO-B levels to 4.52 ± 0.20 ng/mg protein, while ML154 at 0.1 µM further lowered levels to 3.84 ± 0.42 ng/mg protein. At 1 µM ML154, MAO-B levels rose again to 5.61 ± 0.44 ng/mg protein. These findings indicate that NPS reduces dopamine degradation by lowering MAO-B levels, although higher doses of ML154 appear to counteract this effect (Fig. 8B).

COMT levels in the control group were 11.54 ± 0.95 ng/mg protein and remained unchanged with NPS treatment (11.44 ± 0.20 ng/mg protein). Paraquat exposure led to a significant increase in COMT levels to 13.00 ± 0.59 ng/mg protein (*p* < 0.05). Co-treatment with Paraquat + NPS reduced levels to 10.00 ± 0.37 ng/mg protein, while further addition of ML154 at 0.1 µM maintained similar levels (10.17 ± 0.51 ng/mg protein). At 1 µM ML154, COMT levels decreased further to 8.89 ± 0.48 ng/mg protein. These findings suggest that NPS counteracts paraquat-induced increases in COMT levels, and higher doses of ML154 potentiate this effect (Fig. 8C).

TBARS, a marker of lipid peroxidation, was 3.78 ± 0.19 nmol/mg protein in control cells. Paraquat exposure significantly increased TBARS levels to 5.28 ± 0.30 nmol/mg protein, indicating elevated oxidative stress (*p* < 0.05). Co-treatment with Paraquat + NPS reduced TBARS levels to 3.12 ± 0.23 nmol/mg protein, demonstrating the antioxidant effect of NPS. ML154 treatment had a minimal effect on TBARS levels; the Paraquat + NPS + ML154 group exhibited TBARS levels of 3.98 ± 0.10 nmol/mg protein at 0.1 µM and 2.93 ± 0.18 nmol/mg protein at 1 µM. These findings suggest that NPS reduces lipid peroxidation induced by paraquat, with ML154 showing only a modest impact on TBARS levels (Fig. 8D).

### Dopamine Levels in d-SH-SY5Y Cells

**Fig. 9:**
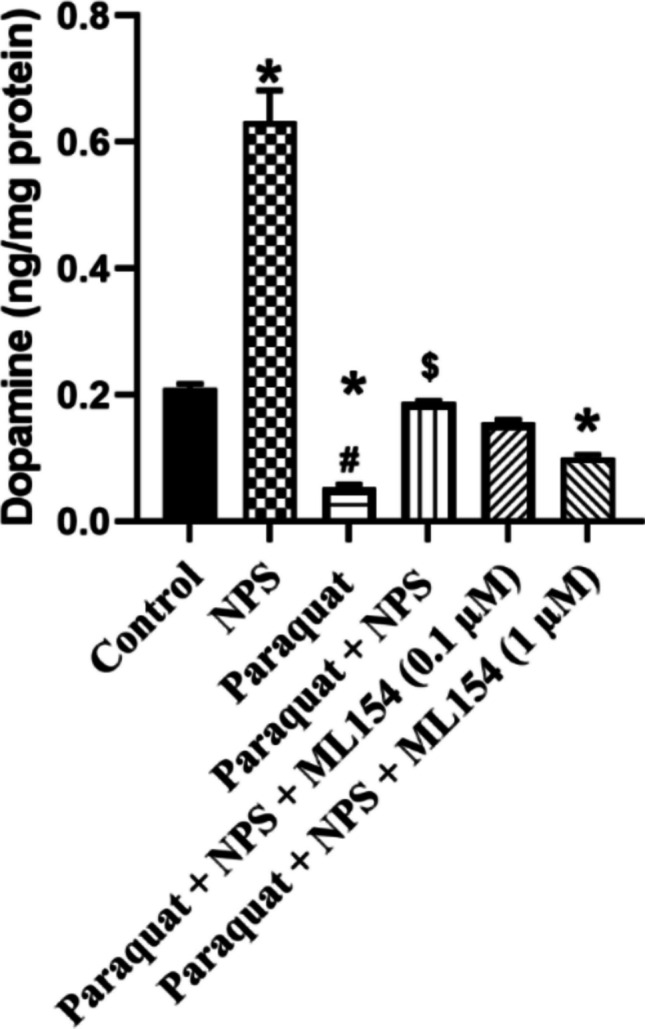
Dopamine levels measured in d-SH-SY5Y cell homogenates. *p < 0.05, compared with control, ^#^*p* < 0.05, compared with NPS, ^$^*p* < 0.05, compared with Paraquat-treated group (*n* = 8)

Control cells exhibited dopamine levels of 0.21 ± 0.005 ng/mg protein, which increased to 0.64 ± 0.049 ng/mg protein with NPS treatment, indicating a more than threefold elevation. Paraquat treatment drastically reduced dopamine levels to 0.053 ± 0.004 ng/mg protein. Co-treatment with Paraquat + NPS restored dopamine levels to 0.18 ± 0.003 ng/mg protein. When combined with ML154, dopamine levels were 0.15 ± 0.004 ng/mg protein at 0.1 µM ML154 and 0.1 ± 0.005 ng/mg protein at 1 µM ML154. These results demonstrate that NPS partially reverses paraquat-induced dopamine depletion, though the NPS antagonist ML154 diminishes this protective effect in a dose-dependent manner (Fig. 9).

### Gene Expression Levels of Apoptotic and Neurotransmitter-Related Proteins in d-SH-SY5Y Cells

**Fig. 10:**
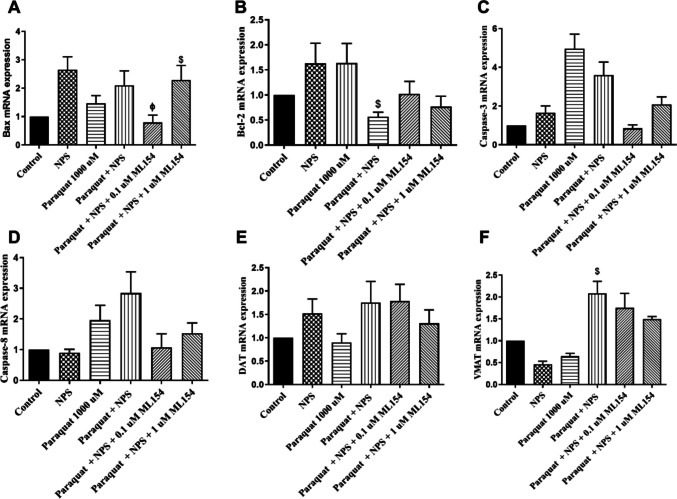
Measured (**A**) Bax, (**B**) Bcl-2, (**C**) Caspase-3, (**D**) Caspase-8, (**E**) DAT, and (**F**) VMAT mRNA levels in d-SH-SY5Y cells. (^$^*p* < 0.05, compared with Paraquat^*,* ϕ^*p* < 0.05, compared with Paraquat + NPS-treated group) (*n* = 7 for Bax, *n* = 5 for Bcl-2, *n* = 3 for Caspase-3, Caspase-8, DAT, and VMAT)

- **Bax mRNA Expression:** Paraquat slightly elevated Bax expression, but the increase was not significant compared to control (*p* > 0.05). Other treatments had no significant impact on Bax expression (Fig. 10A).
- **Bcl-2 mRNA Expression:** Paraquat exposure had no significant effect on Bcl-2 expression compared to control (*p* > 0.05). Co-treatment with Paraquat + NPS significantly increased Bcl-2 expression compared to only Paraquat treatment. Addition of ML154 (for all concentrations) to the Paraquat + NPS group did not create a statistically significant difference (Fig. 10B).
- **Caspase-3 and Caspase-8 mRNA Expression:** Paraquat increased caspase 3 mRNA expression compared to control, while the levels decreased in the group treated with Paraquat + NPS. A slight increase in caspase 8 mRNA expression was observed with paraquat application compared to control, and also observed when paraquat was applied together with NPS. However, none of these changes reached statistical significance (*p* > 0.05) (Fig. 10C, D).
- **DAT and VMAT mRNA Expression:** DAT expression was reduced by paraquat compared to control, but changes across groups were not statistically significant (*p* > 0.05). The addition of NPS to paraquat resulted in a slight increase in DAT mRNA expression compared to paraquat treatment alone; however, this difference was not statistically significant (*p* > 0.05) (Fig. 10E). VMAT mRNA expression decreased with paraquat application compared to control, but it did not reach statistical significance. However, paraquat + NPS application increased VMAT mRNA expression significantly compared to only paraquat treatment (*p* < 0.05; Fig. 10F).

### Immunofluorescence Analysis

**Fig. 11:**
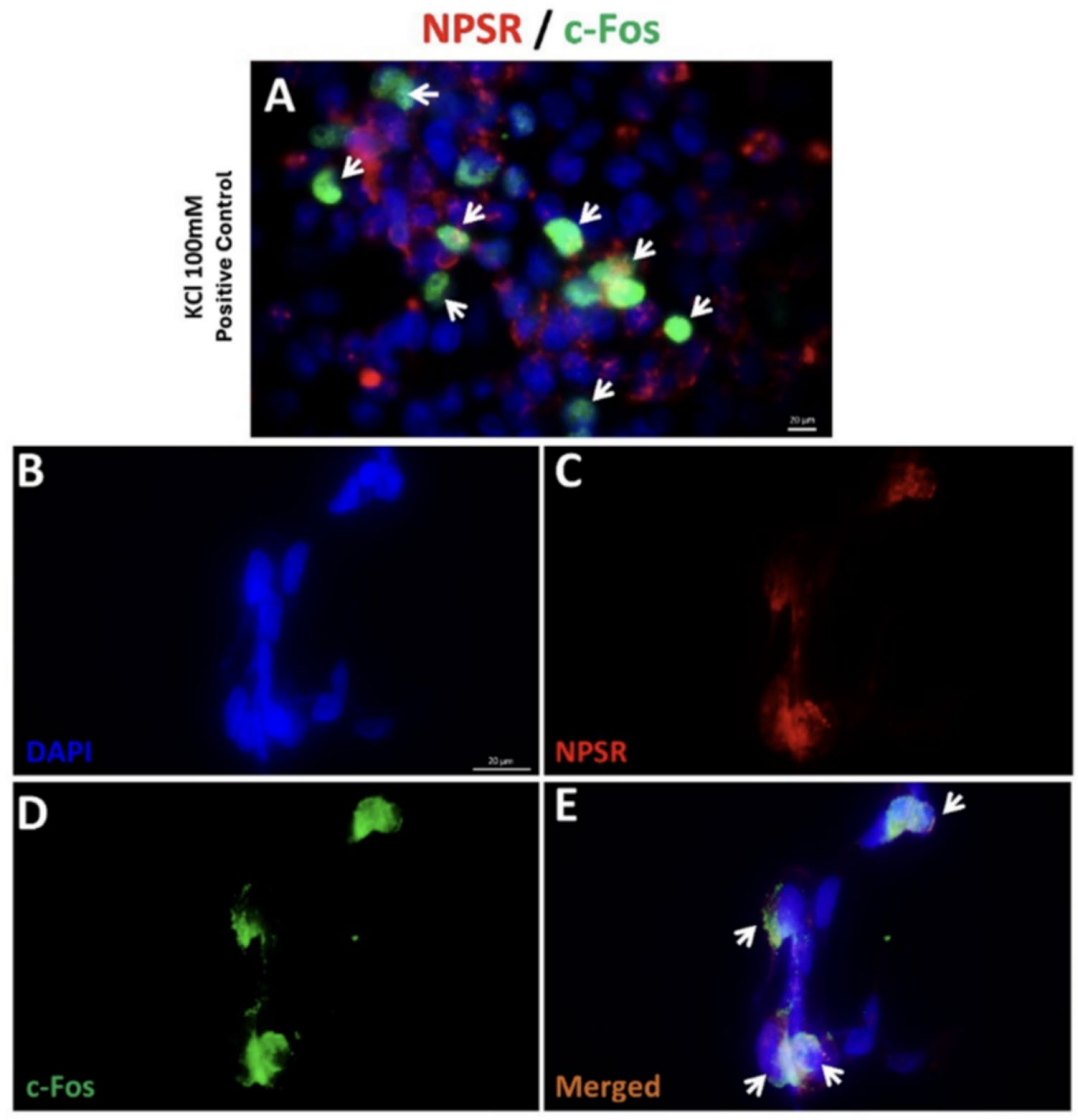
Immunofluorescence staining of c-Fos (green fluorescence, white arrows) in NPSR + (red fluorescence) d-SH-SY5Y cells treated with KCl, used as a positive control. **A** Merged image showing DAPI-stained nuclei (blue), NPSR (red), and c-Fos (green) signals; white arrows indicate cells positive for c-Fos expression. **B** DAPI staining of nuclei. **C** Immunofluorescence staining for NPSR. **D** Immunofluorescence staining for c-Fos. **E** Merged image of DAPI, NPSR, and c-Fos signals; white arrows highlight co-localization of c-Fos expression in NPSR⁺cells. Scale bars: 20 µm

To confirm functional neuronal activation in NPSR-expressing differentiated SH-SY5Y cells, 100 mM KCl was applied as a depolarizing agent. Following treatment, a marked increase in c-Fos immunoreactivity was observed in NPSR⁺ cells (Fig. 11A), indicating that the cells are capable of activity-dependent gene expression. High co-localization of NPSR (red) (Fig. 11C) with c-Fos (green) (Fig. 11D) signals in merged images (Fig. 11E) supports the activation of NPSR⁺ neurons upon depolarization. These findings validate the responsiveness of the neuronal population and confirm that NPSR-expressing cells can be functionally activated under appropriate stimuli.

**Fig. 12:**
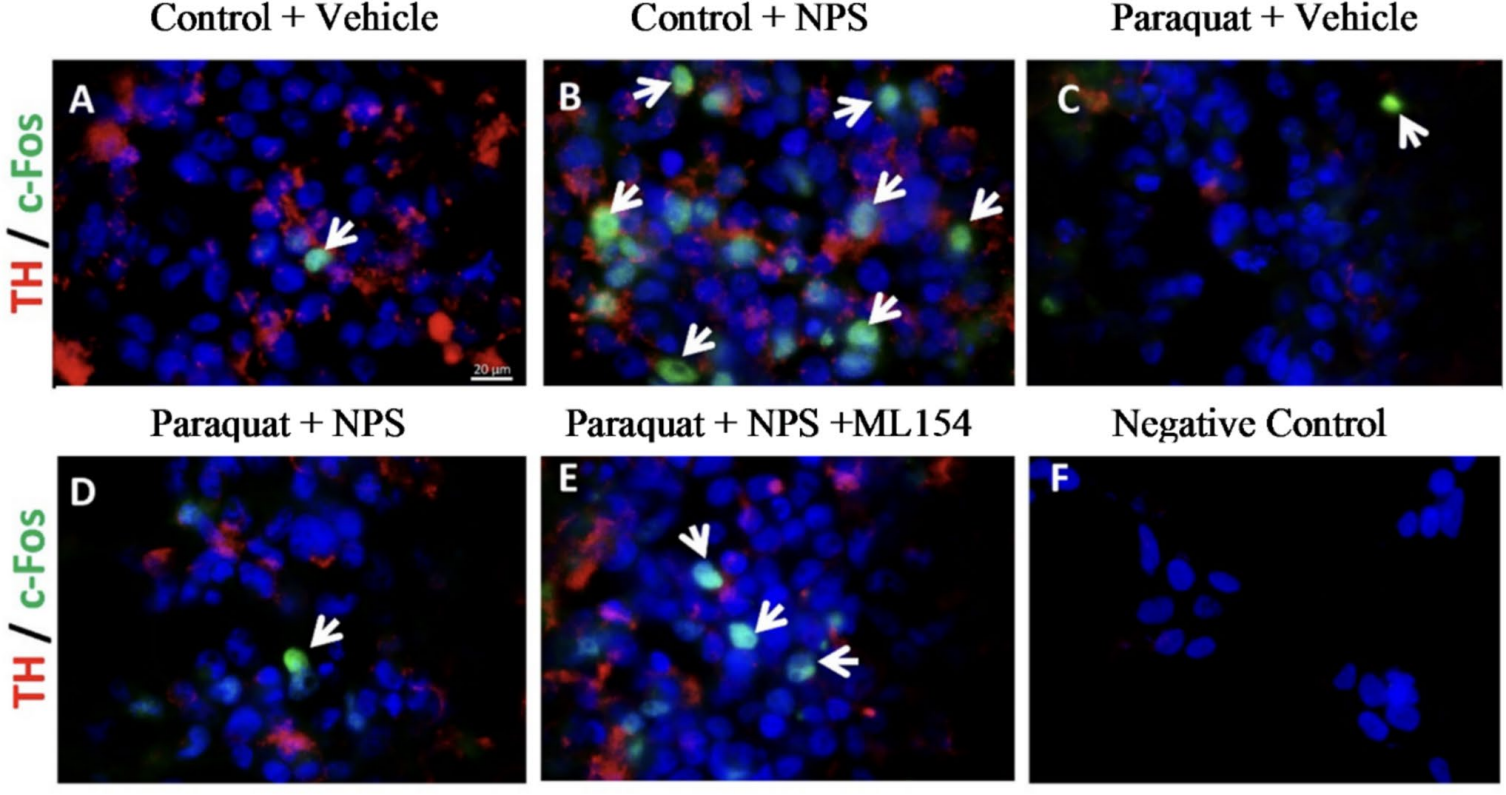
Immunofluorescence staining of TH (red) and c-Fos (green, white arrows) in control + vehicle (**A**), control + NPS (**B**), Paraquat + vehicle (**C**), Paraquat + NPS (**D)**, Paraquat + NPS + ML154 (**E**), and negative control (**F**) (Blue; DAPI)

In the control + vehicle group, moderate TH expression was observed along with sparse c-Fos-positive cells (Fig. 12A). NPS treatment alone (control + NPS) markedly increased the number of c-Fos-immunoreactive cells, suggesting enhanced neuronal activation (Fig. 12B). In contrast, paraquat exposure led to a clear reduction in TH signal intensity, consistent with dopaminergic neuronal damage, and only minimal c-Fos expression was detected (Fig. 12C). However, co-treatment with Paraquat + NPS partially restored TH expression and significantly increased c-Fos immunoreactivity compared to the Paraquat + vehicle group (Fig. 12D), indicating a neuroprotective and activating role of NPS under oxidative stress conditions. Notably, the addition of the NPSR antagonist ML154 (Paraquat + NPS + ML154) attenuated this effect, as seen by a reduction in c-Fos-positive cells (Fig. 12E), suggesting that the observed activation is mediated via NPSR-dependent mechanisms. The negative control showed no specific immunoreactivity (Fig. 12F).

## Discussion

PD is a progressive neurodegenerative disorder primarily characterized by the selective degeneration of dopaminergic neurons in the substantia nigra. This neuronal loss leads to the hallmark motor symptoms of the disease, including bradykinesia, rigidity, resting tremor, and postural instability [34, 35]. Although the precise molecular underpinnings of PD remain partially unresolved, converging evidence implicates both genetic susceptibility and environmental toxins in its pathogenesis. Among these, oxidative stress and mitochondrial dysfunction are recognized as critical contributors to dopaminergic vulnerability.

In this context, paraquat, a widely used herbicide with structural similarity to the neurotoxin MPP⁺, has been extensively utilized to model PD both in vivo and in vitro. Paraquat accumulates in dopaminergic neurons, and initiates neurotoxicity primarily through redox cycling, which leads to excessive ROS generation, mitochondrial impairment, and subsequent activation of apoptotic pathways [36–39]. Additionally, paraquat impairs endogenous antioxidant defenses, enhances lipid peroxidation, disrupts dopamine metabolism, and triggers the expression of pro-apoptotic mediators—collectively mimicking the pathophysiological features of PD [40–42].

Emerging evidence suggests that NPS may exert neuroprotective effects. NPS signaling is known to regulate intracellular calcium levels and modulate several downstream cascades, including those linked to arousal, anxiety, and cellular stress [43–45]. Studies extend its functional repertoire, indicating that NPS can counteract oxidative stress, stabilize dopamine levels, and suppress apoptotic signals under neurotoxic conditions [13, 46–48].

In our experimental design utilizing SH-SY5Y cells—a well-established dopaminergic-like neuronal model—we investigated the potential neuroprotective effects of NPS against paraquat-induced neurotoxicity. To establish a PD-like in vitro model, SH-SY5Y neuroblastoma cells were first differentiated using RA, followed by exposure to paraquat, a widely recognized environmental neurotoxin that selectively impairs dopaminergic neurons. Differentiation significantly enhanced the expression of neuronal markers such as TH and PGP9.5, whereas subsequent paraquat treatment led to a marked downregulation of these markers, along with pronounced neurite retraction and reduced cell viability—collectively confirming the successful induction of a PD-like neurodegenerative phenotype. Importantly, the dose and duration of paraquat exposure play a critical role in determining the extent of neurotoxicity. In previous studies utilizing PC12 neuronal cells, paraquat has been applied at concentrations ranging from 200 to 1000 µM, and the dose employed in our study falls within this established range [49]. This model builds upon our previous work, in which we systematically optimized key culture parameters—including collagen coating, fetal bovine serum (FBS) concentration, differentiation agents, and neurotoxin application—to improve the consistency and physiological relevance of SH-SY5Y-based PD models [21]. Notably, central administration of NPS has been shown to ameliorate these motor deficits and restore TH-positive neuronal populations in parkinsonian rat models [50]. Integrating these insights, our in vitro system provides a refined and mechanistically informative platform to explore the intracellular pathways involved in paraquat-induced neurotoxicity and to evaluate the neuroprotective potential of candidate compounds such as NPS.

Before delving into the molecular alterations induced by paraquat and the restorative effects of NPS, it is important to evaluate the overall impact of paraquat exposure on cellular energy metabolism and mitochondrial function, as these parameters are crucial determinants of neuronal viability and function. In our study, treatment with 1000 µM paraquat for 24 h led to a marked reduction in intracellular ATP content in d-SH-SY5Y cells, reflecting a pronounced impairment of mitochondrial energy metabolism. These findings are consistent with previous reports demonstrating that paraquat disrupts mitochondrial homeostasis by accumulating within mitochondria in a membrane potential-dependent manner and undergoing rapid redox cycling to generate superoxide and other reactive oxygen species [51–53]. The mono-cation radical form of paraquat reacts with molecular oxygen, initiating a cascade of oxidative damage that compromises the mitochondrial membrane potential (MMP), destabilizes inner membrane integrity, and impairs the function of electron transport chain complexes, particularly Complex I and Complex III [54, 55]. The resulting bioenergetic failure reduces ATP synthesis and activates mitochondria-dependent apoptotic signaling, including cytochrome c release and caspase activation [56, 57]. Structural alterations such as mitochondrial swelling, cristae disruption, and increased lipid peroxidation have also been observed within similar exposure windows [58, 59]. These mechanistic insights provide a robust framework for interpreting our observed declines in ATP level as reliable markers of mitochondrial dysfunction and oxidative stress.

Importantly, our data further show that co-treatment with NPS significantly ameliorated paraquat-induced mitochondrial deficits. These findings suggest a potential protective role of NPS in preserving mitochondrial bioenergetics, possibly through modulation of redox balance or preservation of mitochondrial structure. However, the dose-dependent reversal of this protective effect by ML154 implies that NPS-mediated mitochondrial protection is receptor-dependent. To date, direct evidence linking NPS to mitochondrial ATP production remains limited, highlighting a critical gap in the literature. Given that other neuropeptides have been shown to regulate mitochondrial dynamics and oxidative metabolism, further mechanistic studies are warranted to elucidate the signaling pathways through which NPS influences mitochondrial energy homeostasis.

At the molecular level, paraquat significantly increased ERK1/2 phosphorylation—indicative of cellular stress—and suppressed Nrf2 expression, a master regulator of antioxidant responses. Nrf2 typically functions via the antioxidant response element (ARE), modulating genes involved in redox balance and cytoprotection. Under stress-free conditions, Nrf2 remains bound to Keap1 in the cytoplasm, but upon oxidative insult, it dissociates and translocates to the nucleus [60–62]. Nrf2 signaling is impaired in aging and neurodegenerative diseases, including PD [63, 64], and its deficiency exacerbates oxidative damage and iron accumulation in the substantia nigra [65]. In line with these findings, our study demonstrated that paraquat treatment resulted in a marked reduction of Nrf2 expression in the neuronal cells [49]. Our findings confirm that paraquat suppresses Nrf2 expression, while NPS treatment restores it—suggesting that NPS bolsters antioxidant defense by reactivating Nrf2.

Consistent with PD pathology, paraquat-induced oxidative stress was evident by elevated TBARS levels, indicative of increased lipid peroxidation. These results are in agreement with prior studies reporting enhanced TBARS, 4-HNE, and MDA in oxidative stress conditions related to PD [66–69]. NPS co-treatment significantly reduced TBARS levels, aligning with its known antioxidant activity in the substantia nigra and hippocampus [46, 70, 71]. While ML154 partially reversed this effect, the limited impact at higher concentrations may point to both receptor-dependent and -independent mechanisms.

We also examined how paraquat and NPS influence dopamine metabolism, focusing on key enzymes such as MAO-A, MAO-B, and COMT. Paraquat significantly elevated MAO-B and COMT levels—both of which degrade dopamine and contribute to ROS production—thereby exacerbating dopaminergic dysfunction. These alterations closely resemble those observed in PD and aging models [72]. Notably, selegiline (also known as deprenyl), a selective and irreversible MAO-B inhibitor, is clinically employed in PD treatment to enhance dopamine availability and attenuate oxidative stress associated with its metabolism [73]. In this context, the ability of NPS to reduce MAO-B and COMT expression suggests its potential role in preserving dopamine levels and limiting oxidative damage. Interestingly, co-treatment with low-dose ML154 enhanced NPS-induced MAO-B suppression, while higher doses reversed this effect, indicating a complex dose-dependent regulation possibly involving compensatory or feedback mechanisms. In contrast, paraquat reduced MAO-A levels, likely reflecting mitochondrial dysfunction and selective neuronal vulnerability. NPS treatment partially restored MAO-A expression, an effect that was also potentiated by low-dose ML154, further supporting the involvement of NPSR-dependent intracellular pathways in modulating dopamine metabolism. Collectively, these findings highlight the multifaceted role of NPS in regulating dopamine homeostasis under neurotoxic stress.

Regarding intracellular dopamine content, paraquat treatment led to a substantial (~ 75%) reduction, consistent with PD pathology and prior findings on paraquat’s dopaminergic toxicity. Notably, NPS treatment not only prevented dopamine depletion but also increased dopamine levels above basal control levels. This effect may result from enhanced dopamine synthesis, reduced degradation, or protection of dopaminergic terminals. A previous study support such trophic effects of NPS via calcium signaling and neuroprotective pathways [45]. However, the restorative effect of NPS was diminished by ML154 in a dose-dependent manner, supporting the receptor-mediated mechanism and reinforcing the therapeutic relevance of the NPS/NPSR axis in dopaminergic neuroprotection.

Apoptotic markers were also examined. Paraquat slightly increased Bax mRNA expression, indicative of a shift toward mitochondrial apoptosis. While Bcl-2 levels remained unchanged with paraquat alone, NPS treatment significantly upregulated Bcl-2, consistent with activation of survival pathways like PI3K/Akt or ERK [74]. Interestingly, this upregulation was not fully reversed by ML154, suggesting partial receptor independence.

Caspase-3 expression increased following paraquat exposure, a result in line with ROS-induced apoptosis [75], and was attenuated by NPS. Caspase-8 levels remained relatively stable, suggesting that paraquat predominantly activates the intrinsic, rather than extrinsic, apoptotic pathway, consistent with prior reports [20, 76].

DAT and VMAT are critical components of dopaminergic neurotransmission, responsible for dopamine reuptake and vesicular storage, respectively. Alterations in their expression can reflect or contribute to dopaminergic dysfunction, especially under toxic insults such as paraquat exposure.

Although paraquat treatment led to a reduction in DAT mRNA expression compared to control, the change did not reach statistical significance. Interestingly, co-treatment with NPS resulted in a modest increase in DAT levels compared to paraquat alone, suggesting a potential trend toward the restoration of dopaminergic tone. However, this change was also not statistically significant. Previous studies have shown that the absence or inhibition of DAT can reduce paraquat-induced neurotoxicity, likely by limiting intracellular uptake of the toxin [68]. These findings suggest that paraquat’s neurotoxic effects may not rely solely on DAT-mediated uptake but that DAT may still play a modulatory role in determining neuronal vulnerability.

VMAT, which sequesters cytosolic dopamine into synaptic vesicles, plays a key role in protecting neurons from dopamine-induced oxidative stress. In our study, paraquat exposure resulted in a decrease in VMAT mRNA expression, although this was not statistically significant. Importantly, co-treatment with NPS significantly increased VMAT expression compared to paraquat alone. This upregulation of VMAT suggests that NPS may enhance the vesicular storage of dopamine, thereby limiting cytosolic dopamine accumulation — a major contributor to oxidative damage in dopaminergic neurons. Given that impaired vesicular storage is associated with increased dopamine autoxidation and oxidative stress, the NPS-mediated preservation of VMAT expression may represent a protective mechanism for maintaining dopaminergic neuron integrity under toxic conditions.

Neuronal activation is often accompanied by rapid and transient expression of immediate early genes such as c-Fos, which serves as a molecular marker of activity-dependent gene expression and is tightly regulated by intracellular calcium signaling pathways [77, 78]. In our study, depolarization with high-potassium (100 mM KCl) treatment significantly increased c-Fos immunoreactivity in NPS receptor-expressing (NPSR⁺) differentiated SH-SY5Y cells. The strong co-localization of c-Fos and NPSR signals confirms that these cells are functionally responsive to depolarizing stimuli, thereby validating their physiological activation capacity in vitro.

Under basal conditions, c-Fos expression was minimal, and moderate TH expression indicated a dopaminergic phenotype. However, exogenous NPS treatment markedly increased the number of c-Fos-positive cells, suggesting that NPS can directly stimulate neuronal activation via NPSR in dopaminergic-like SH-SY5Y cells. This finding is consistent with our previous in vivo results, in which central NPS administration induced c-Fos activation in NPSR1-expressing neurons in the substantia nigra of rats and mice [50], further supporting the functional relevance of the NPS–NPSR system in dopaminergic regions of the brain.

Exposure to paraquat resulted in a pronounced decline in both TH and c-Fos immunoreactivity, consistent with dopaminergic neuronal damage and impaired activity-dependent transcription. Notably, co-treatment with NPS not only restored TH expression but also significantly enhanced c-Fos levels compared to paraquat alone, suggesting that NPS exerts both neuroprotective and stimulatory effects under oxidative stress conditions. Importantly, these effects were significantly diminished by the NPSR antagonist ML154, reinforcing that the observed activation is mediated via NPSR-dependent mechanisms.

In the current study, the cellular calcium level was not evaluated, nor did we perform calcium imaging following the application of ligand; however, it highly appears that the applied NPS induces cellular downstream effects. In fact, NPS signaling has been found to induce calcium mobilization in rodent neuronal cells. It has been reported that once NPSR1 is activated, neuronal calcium transients are observed through mediation of phospholipase C, in addition to the inositol-3-phosphate, and ryanodine receptors [45, 79]. Moreover, using singlecell calcium imaging, Erdman found that NPSR1-induced calcium mobilization from the endoplasmic reticulum via activation of IP3 and ryanodine receptors in primary hippocampal cultures [45]. Additionally, Park and colleagues recently showed in mice brain slice preparations that NPS-induced activation of GPR154 causes an increase in neuronal excitability through calcium ions which seems to be involved both with Gαq signaling and inhibition of the voltage-gated potassium channels [80].

In rodent studies, the selective NPSR antagonist, ML154 was reported to suppress the action both endogenous and exogenously administered NPS [28, 81]. More importantly, application of ML154 has been shown to inhibit NPS-induced stimulation of cellular calcium, cAMP, and ERK phosphorylation responses with IC50 values of 36.5 nM, 22.1 nM, and 9.3 nM, respectively [82].

Mechanistically, NPSR activation initiates intracellular signaling cascades involving calcium mobilization and ERK1/2 phosphorylation, both of which are known regulators of c-Fos expression and neuronal gene programs [83]. Although early paraquat exposure may not always elevate ERK1/2 [84], in our study, paraquat exposure significantly increased ERK1/2 phosphorylation, likely reflecting dose- and time-dependent cellular stress dynamics, while NPS treatment effectively reversed this increase. Upon binding of NPS, NPSR1 signals primarily through Gq-coupled pathways, triggering IP₃ production and subsequent release of calcium from intracellular stores, followed by store-operated calcium entry [45, 85, 86]. The critical role of these signaling events is further supported by Batran et al. (2021), who showed that NPS exhibits potent agonistic activity for both calcium (EC₅₀ = 0.4 µM) and cAMP (EC₅₀ = 1.84 µM) pathways, while ML154 effectively blocks both signals at submicromolar IC₅₀ values [80].

These findings suggest that ML154 blocks the pathways through which NPS exerts its antioxidant and antiapoptotic effects, including ERK1/2 phosphorylation and Nrf2 upregulation, as demonstrated in our study. Therefore, the administration of ML154 in our experiments provides mechanistic confirmation that the observed cellular effects are indeed mediated by receptor-specific activation of NPSR1 and its downstream Ca^2^⁺- and cAMP-dependent pathways. Furthermore, this dual inhibitory capacity strengthens the notion that NPS effects are tightly coupled to receptor-mediated calcium dynamics. Also, the significant increase in c-Fos expression following NPS treatment—and its suppression by ML154—serves as indirect yet strong functional evidence for NPS-induced calcium signaling.

Taken together, while we did not directly measure intracellular calcium levels, the combined evidence of ERK1/2 modulation, Nrf2 activation, c-Fos upregulation, and ML154 sensitivity strongly supports the hypothesis that NPS activates calcium-sensitive intracellular signaling pathways via NPSR1. Future studies using calcium imaging or fluorescent Ca^2^⁺ indicators would be valuable to directly confirm these mechanistic insights.

To support these receptor-specific findings from a molecular perspective, we also employed a curated bioinformatic approach. In addition to the pharmacological blockade of NPSR1 with ML154, the bioinformatic evidence from the Reactome database provides a curated molecular validation of NPS–NPSR1 binding. The identification of this receptor–ligand event (R-HSA-444620) and its integration within a broader GPCR signaling framework (R-HSA-373076) support the hypothesis that the neuroprotective effects of NPS are primarily receptor-dependent. The downstream activation of intracellular signaling cascades—particularly calcium mobilization and ERK phosphorylation—has been previously implicated in cell survival and synaptic plasticity, which align with the observed functional outcomes in our experimental model. Thus, bioinformatic validation not only reinforces the specificity of the NPS–NPSR1 interaction but also strengthens the translational relevance of targeting this pathway in neurodegenerative conditions. To further strengthen this interpretation, three-dimensional docking simulations were conducted to visualize the structural interface of the NPS–NPSR1 complex. The analysis revealed a network of stabilizing non-covalent interactions within the binding pocket, including multiple hydrogen bonds, salt bridges, and π–π stacking interactions. These molecular contacts are indicative of a high-affinity and specific ligand–receptor binding profile, supporting the functional data demonstrating NPSR1-dependent activity. Collectively, the integration of pharmacological, bioinformatic, and structural data provides a robust validation of the NPS–NPSR1 interaction and highlights its potential as a therapeutic target in neurodegenerative conditions.

When examining these receptor-specific effects, dose-dependent results should also be taken into account. Interestingly, our findings revealed a dose-dependent paradox in the effects of the NPSR1 antagonist ML154. While low-dose ML154 (0.1 µM) appeared to partially potentiate the neuroprotective actions of NPS—such as the downregulation of MAO-B and COMT, restoration of MAO-A, and upregulation of Bcl-2—higher concentrations (1 µM) attenuated or reversed these beneficial effects. This paradoxical response may reflect differential modulation of receptor signaling at varying antagonist concentrations. At low doses, ML154 may act as a partial antagonist or allosteric modulator, subtly altering receptor conformation and enhancing selective downstream signaling. In contrast, higher doses could induce full antagonism or receptor desensitization, thereby suppressing NPS-mediated cytoprotective signaling pathways, including ERK1/2 and Nrf2.

Similar biphasic effects of receptor antagonists have been reported in other G protein-coupled receptor systems, where ligand-receptor interaction dynamics and downstream signaling profiles may vary depending on ligand concentration, receptor occupancy, and cellular context [87–89]. It is also plausible that higher concentrations of ML154 exert off-target or compensatory effects, thereby disrupting the tightly regulated oxidative and dopaminergic balance maintained by NPS. Therefore, careful titration of ML154 concentration is crucial when delineating receptor-specific mechanisms, and our findings underscore the importance of considering dose–response relationships when interpreting pharmacological blockade outcomes.

These dose-dependent outcomes also support the hypothesis that NPSR1 signaling may be subject to functional selectivity (biased agonism), where different levels of receptor engagement elicit distinct downstream responses. Further studies employing genetic knockdown or alternative antagonists may help clarify this complex regulatory dynamic and validate the therapeutic window for targeting the NPS/NPSR system in PD and related disorders.

Taken together, these findings demonstrate that NPS confers potent neuroprotection against paraquat-induced dopaminergic neurotoxicity in an in vitro model of PD. NPS mitigates oxidative stress by restoring Nrf2 signaling and reducing lipid peroxidation, preserves intracellular dopamine levels by downregulating dopamine-catabolizing enzymes (MAO-B and COMT), and promotes dopaminergic neuron survival through upregulation of anti-apoptotic Bcl-2 and suppression of pro-apoptotic caspase-3 expression. Moreover, NPS modulates key components of dopaminergic neurotransmission, including DAT and VMAT, thereby supporting synaptic dopamine homeostasis. The majority of these protective effects are mediated via the NPS receptor, as evidenced by the dose-dependent attenuation following ML154 co-treatment.

**Fig. 13:**
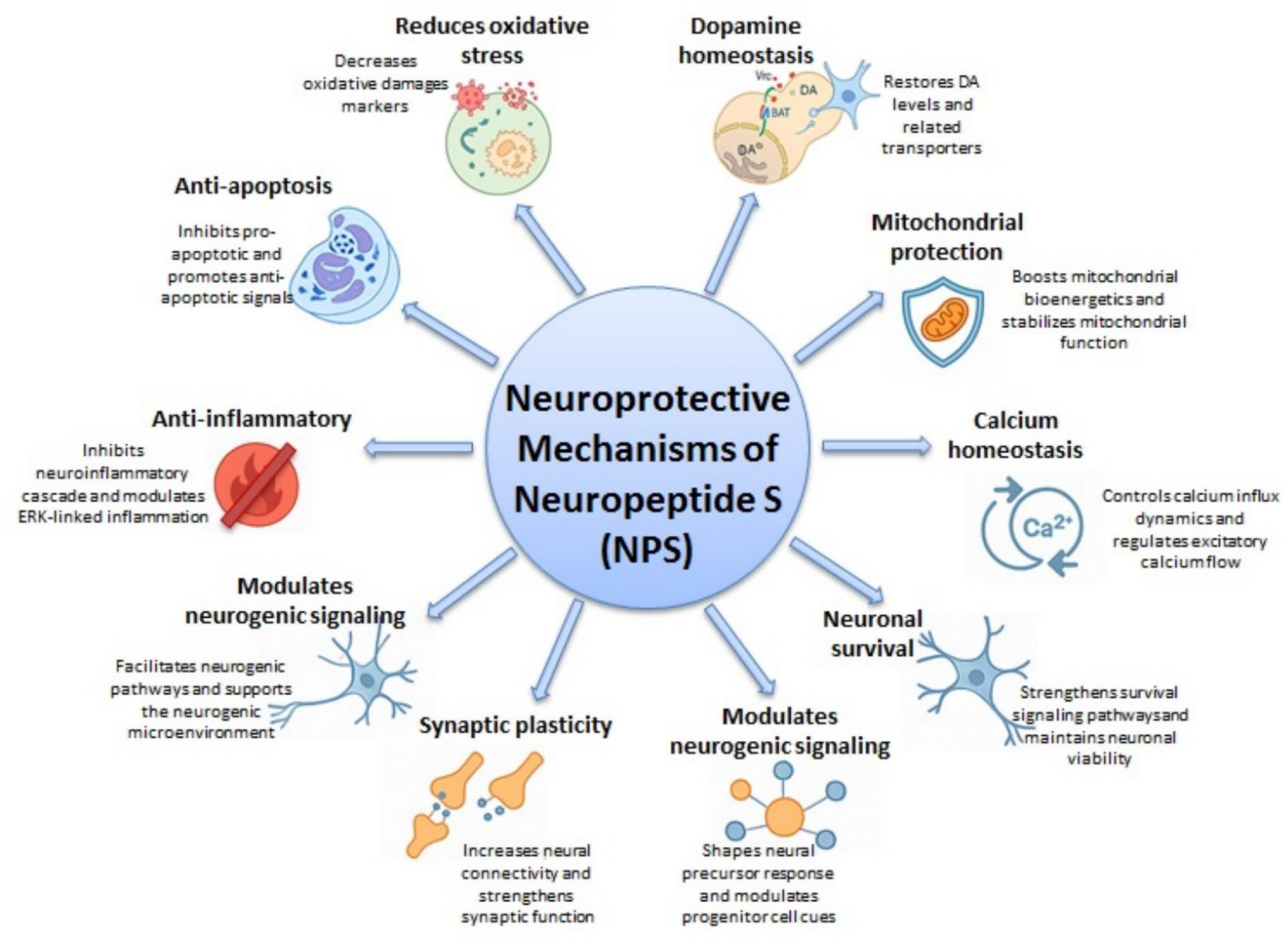
Proposed schematic illustration of the neuroprotective mechanisms of NPS

Figure 13 provides a schematic illustration of the proposed neuroprotective mechanisms of NPS. This integrative figure combines the experimental findings from our cellular model with relevant evidence from in vivo studies reported in the literature. By summarizing the convergent molecular pathways—including modulation of oxidative stress, mitochondrial preservation, dopaminergic regulation, and anti-apoptotic signaling—the figure highlights how NPS exerts multifaceted neuroprotective effects. Placing our results within this broader mechanistic framework allows a clearer understanding of the translational relevance of NPS in Parkinson’s disease models.

## Conclusion

This study provides evidence in a cellular PD model that NPS exerts neuroprotective effects, though further in vivo studies are necessary to confirm these findings. Through the activation of the ERK/Nrf2 signaling axis and NPS receptor-mediated pathways, under our single-dose, short-term conditions, NPS alleviated oxidative stress, preserved dopamine levels, and modulated apoptosis-related gene expression. Further dose–response and long-term studies are needed to substantiate these findings. The observed upregulation of the anti-apoptotic gene Bcl-2, along with the downregulation of pro-apoptotic markers Bax, caspase-3, and caspase-8, underscores its capacity to promote neuronal survival under oxidative stress conditions. Importantly, NPS significantly downregulated the expression levels of MAO-A and MAO-B -key enzymes involved in dopamine catabolism—suggesting that NPS may contribute to dopaminergic neuroprotection by reducing dopamine degradation and subsequent ROS generation. Additionally, the ability of NPS to regulate dopaminergic transporter genes such as DAT and VMAT, and to restore the expression of TH, further supports its role in maintaining dopaminergic homeostasis. To the best of our knowledge, this is the first study to provide such an in-depth analysis of the intracellular molecular mechanisms modulated by NPS under neurodegenerative conditions, revealing its multifaceted neuroprotective profile. Collectively, these findings indicate that the NPS/NPSR signaling system could represent a promising target in Parkinson’s disease, although further studies are needed to establish its translational relevance. Future in vivo studies are warranted to confirm these neuroprotective mechanisms and assess the translational applicability of NPS-based interventions.

## Limitations and Future Directions

Despite the promising findings of this study, several limitations should be acknowledged. First, the use of SH-SY5Y cells as a model for PD, while widely accepted, does not fully replicate the complexity of dopaminergic neurons in vivo. Future studies should aim to validate these findings in more complex in vitro models, such as primary dopaminergic neurons or 3D organoids, to better mimic the pathological conditions of PD. Second, our study was limited to single-dose, short-term exposure, and therefore the dose–response relationship and long-term effects of NPS remain unexamined. Further investigations into the chronic administration of NPS in animal models of PD are necessary to assess its therapeutic potential and safety profile.

Moreover, some of the observed effects, such as changes in Bax, Caspase-8, and DAT expression, did not reach statistical significance and should be interpreted with caution. These non-significant findings highlight the need for replication in larger-scale studies. In addition, although calcium signaling has been reported as a downstream mediator of NPS in previous literature, intracellular calcium levels were not directly measured in the present study, representing another limitation. Unexpected dose-dependent effects of ML154 were also observed, suggesting possible off-target or complex receptor interactions, which warrant further investigation.

Finally, while our study focuses on specific signaling pathways, it is important to recognize that neuroprotection is likely mediated through multiple interconnected mechanisms. Future research should not only further explore the pathways identified in our study but also investigate other neurodegenerative processes that may contribute to the overall therapeutic effects of NPS. Expanding the scope of research to include additional molecular targets and cellular processes could provide a more comprehensive understanding of how NPS exerts its neuroprotective effects. Ultimately, clinical studies will be required to determine the efficacy of NPS as a potential treatment for PD in human patients. In conclusion, while our study lays the groundwork for understanding NPS’s neuroprotective mechanisms, further research is essential to fully characterize its therapeutic potential and explore its broader applicability in treating neurodegenerative diseases.

## Data Availability

The data that support the findings of this study are available from the corresponding author upon reasonable request.
